# Complete genome sequence of the hyperthermophilic archaeon *Saccharolobus islandicus* Y08.82.36 isolated from a Yellowstone hot spring

**DOI:** 10.1128/mra.00626-25

**Published:** 2025-07-28

**Authors:** Changyi Zhang, Edward E. Andrews, Thomas W. Cowell, Nikki H. Gedrimas, Rachel J. Whitaker

**Affiliations:** 1 Carl R. Woese Institute for Genomic Biology, University of Illinois Urbana-Champaign 14589, Urbana, Illinois, USA; 2 Department of Microbiology, University of Illinois Urbana-Champaign 14589, Urbana, Illinois, USA; Portland State University 6685, Portland, Oregon, USA

**Keywords:** *Saccharolobus islandicus*, complete genome sequence, viral host

## Abstract

Members of the order Sulfolobales are known to thrive in acidic, high-temperature hot springs. Here, we report the sequencing and complete genome assembly of a hyperthermophilic archaeon *Saccharolobus islandicus* Y08.82.36, isolated from a hot spring in the Norris Geyser Basin, Yellowstone National Park, USA, in 2008.

## ANNOUNCEMENT

*Saccharolobus islandicus* (formerly *Sulfolobus islandicus*) is an aerobic, hyperthermophilic archaeon that was first isolated by Zillig et al. from a hot spring in Iceland (1). Subsequently, *S. islandicus* has been found in hot springs worldwide, establishing it as one of a few archaeal species suitable for studying population biology and evolutionary genomics (2–4). We previously reported an *S. islandicus* isolate Y08.82.36 that serves as a host for propagating a range of archaeal viruses (5–7) but lacked a complete genome necessary for studying host-virus interactions. Here, we use PacBio and Illumina sequencing to resolve the full genome of this strain.

Strain Y08.82.36 was originally isolated from a hot spring sample collected in 2008 from the Norris Geyser Basin, Yellowstone National Park, USA (44.7276°N, 110.7144°W). Hot spring sediment samples were collected in sterile Falcon tubes and stored at room temperature during transport. For isolation, 1 mL of sample was inoculated into 19 mL of DT medium (8) and incubated at 76°C with shaking for 10 days. Enrichment cultures were serially diluted and plated to obtain a single colony using the double-layer plating approach (9), which was then grown in 1 mL DT medium for 3 days without shaking. This culture was transferred into 19 mL of fresh medium and incubated for another 3 days before preparing frozen stocks for storage at −80 °C in 8% (vol/vol) DMSO. High-molecular-weight (HMW) DNA was obtained using the Nanobind CBB Kit (Pacific Biosciences), and total genomic DNA for Illumina sequencing was extracted from the same culture using the DNeasy Blood & Tissue Kit (Qiagen).

For PacBio sequencing, 1 µg of HMW DNA was sheared to an average fragment length of 10 kb using a Megaruptor 3 (Diagenode) and converted into a library using a SMRTbell Express Template Prep Kit 3.0 (Pacific Biosciences). This library was pooled and sequenced on a SMRT cell 25M on a PacBio Revio. Demultiplexing and circular consensus sequence generation were performed using SMRT Link v13.1, requiring three polymerase passes and a read quality of at least 0.99, yielding 24,276 HiFi reads. Filtlong v0.2.1 (10) was used to retain the best 95% of bases by quality, resulting in 22,778 HiFi reads (average length = 6,115 bp; N50 = 6,534 bp; average read quality = 30.2).

Illumina sequencing libraries were prepared from 150 ng DNA using an Illumina DNA Prep Kit (Illumina) and sequenced using a MiSeq with v2 reagents (paired-end; 2 × 250 bp) to produce 496,069 raw reads that were analyzed with FastQC v0.11.9 (11). Adapter removal and quality filtering were performed using Trimmomatic v0.39 (12), yielding 431,982 reads with an average read quality ≥33.0.

All tools were run using default parameters. HiFi reads were assembled with the Autocycler v0.4.0 pipeline (13). Autocycler subsampled the reads and assembled each independent subset using Canu v2.4 (14), Flye v2.9.5 (15), Miniasm v0.3 (16) and Minipolish v0.1.3 (17), NextDenovo v2.5.2 (18) and NextPolish v1.4.1 (19), and Raven v1.8.3 (20). The assembled genome was polished using the Illumina reads and Polypolish v0.6.0 (21). Read mapping to the polished assembly was validated using Breseq v0.39.0 (22). The assembly produced a single, circular contig with a total size of 2,633,435 bp, 53× (PacBio) and 74× (Illumina) coverage, and an average GC content of 35.33%.

Y08.82.36 was predicted to have 2,973 genes using the NCBI Prokaryotic Genome Annotation Pipeline v6.10 (23), including 47 tRNAs and 3 rRNAs. Analysis by CRISPRCasTyper v1.8.0 (24) identified three arrays associated with a complete Type I-D CRISPR-Cas system containing 20, 34, and 77 spacers and an orphan Type III-B system. Notably, Y08.82.36 lacks a Type I-A CRISPR-Cas system, which is typically present in *S. islandicus* strains.

## Data Availability

The full-genome sequence has been deposited into the NCBI GenBank database under accession number CP194013, BioProject accession number PRJNA1266175, and BioSample accession numbers SAMN48667873 (PacBio) and SAMN48667874 (Illumina). Raw sequence reads were deposited into the NCBI Sequence Read Archive under accession numbers SRR33657382 (PacBio) and SRR33657381 (Illumina).

## References

[1] Zillig W, Kletzin A, Schleper C, Holz I, Janekovic D, Hain J, Lanzendörfer M, Kristjansson JK. 1993. Screening for Sulfolobales, their plasmids and their viruses in icelandic solfataras. Syst Appl Microbiol 16:609–628. doi:10.1016/S0723-2020(11)80333-4

[2] Reno ML, Held NL, Fields CJ, Burke PV, Whitaker RJ. 2009. Biogeography of the Sulfolobus islandicus pan-genome. Proc Natl Acad Sci USA 106:8605–8610. doi:10.1073/pnas.0808945106 19435847 PMC2689034

[3] Guo L, Brügger K, Liu C, Shah SA, Zheng H, Zhu Y, Wang S, Lillestøl RK, Chen L, Frank J, Prangishvili D, Paulin L, She Q, Huang L, Garrett RA. 2011. Genome analyses of Icelandic strains of Sulfolobus islandicus, model organisms for genetic and virus-host interaction studies. J Bacteriol 193:1672–1680. doi:10.1128/JB.01487-10 21278296 PMC3067641

[4] Jaubert C, Danioux C, Oberto J, Cortez D, Bize A, Krupovic M, She Q, Forterre P, Prangishvili D, Sezonov G. 2013. Genomics and genetics of Sulfolobus islandicus LAL14/1, a model hyperthermophilic archaeon. Open Biol 3:130010. doi:10.1098/rsob.130010 23594878 PMC3718332

[5] Bautista MA, Zhang C, Whitaker RJ. 2015. Virus-induced dormancy in the archaeon Sulfolobus islandicus. mBio 6:e02565-14. doi:10.1128/mBio.02565-14 25827422 PMC4453537

[6] Bautista MA, Black JA, Youngblut ND, Whitaker RJ. 2017. Differentiation and structure in Sulfolobus islandicus rod-shaped virus populations. Viruses 9:120. doi:10.3390/v9050120 28534836 PMC5454432

[7] Athukoralage JS, McMahon SA, Zhang C, Grüschow S, Graham S, Krupovic M, Whitaker RJ, Gloster TM, White MF. 2020. An anti-CRISPR viral ring nuclease subverts type III CRISPR immunity. Nature 577:572–575. doi:10.1038/s41586-019-1909-5 31942067 PMC6986909

[8] Zhang C, Cooper TE, Krause DJ, Whitaker RJ. 2013. Augmenting the genetic toolbox for Sulfolobus islandicus with a stringent positive selectable marker for agmatine prototrophy. Appl Environ Microbiol 79:5539–5549. doi:10.1128/AEM.01608-13 23835176 PMC3754178

[9] Deng L, Zhu H, Chen Z, Liang YX, She Q. 2009. Unmarked gene deletion and host-vector system for the hyperthermophilic crenarchaeon Sulfolobus islandicus. Extremophiles 13:735–746. doi:10.1007/s00792-009-0254-2 19513584

[10] Wick RR. 2021. GitHub. Filtlong: quality filtering tool for long reads. Available from: https://github.com/rrwick/Filtlong

[11] Andrews S. 2010. FastQC: a quality control analysis tool for high throughput sequencing data. GitHub. Available from: https://github.com/s-andrews/FastQC

[12] Bolger AM, Lohse M, Usadel B. 2014. Trimmomatic: a flexible trimmer for Illumina sequence data. Bioinformatics 30:2114–2120. doi:10.1093/bioinformatics/btu170 24695404 PMC4103590

[13] Wick RR, Howden BP, Stinear TP. 2025. Autocycler: long-read consensus assembly for bacterial genomes. bioRxiv. doi:10.1101/2025.05.12.653612 PMC12460055 40875535

[14] Nurk S, Walenz BP, Rhie A, Vollger MR, Logsdon GA, Grothe R, Miga KH, Eichler EE, Phillippy AM, Koren S. 2020. HiCanu: accurate assembly of segmental duplications, satellites, and allelic variants from high-fidelity long reads. Genome Res 30:1291–1305. doi:10.1101/gr.263566.120 32801147 PMC7545148

[15] Kolmogorov M, Yuan J, Lin Y, Pevzner PA. 2019. Assembly of long, error-prone reads using repeat graphs. Nat Biotechnol 37:540–546. doi:10.1038/s41587-019-0072-8 30936562

[16] Li H. 2016. Minimap and miniasm: fast mapping and de novo assembly for noisy long sequences. Bioinformatics 32:2103–2110. doi:10.1093/bioinformatics/btw152 27153593 PMC4937194

[17] Wick RR. 2019. Minipolish: a tool for racon polishing of miniasm assemblies. GitHub. Available from: https://github.com/rrwick/Minipolish

[18] Hu J, Wang Z, Sun Z, Hu B, Ayoola AO, Liang F, Li J, Sandoval JR, Cooper DN, Ye K, Ruan J, Xiao C-L, Wang D, Wu D-D, Wang S. 2024. NextDenovo: an efficient error correction and accurate assembly tool for noisy long reads. Genome Biol 25:107. doi:10.1186/s13059-024-03252-4 38671502 PMC11046930

[19] Hu J, Fan J, Sun Z, Liu S. 2020. NextPolish: a fast and efficient genome polishing tool for long-read assembly. Bioinformatics 36:2253–2255. doi:10.1093/bioinformatics/btz891 31778144

[20] Vaser R, Šikić M. 2021. Time- and memory-efficient genome assembly with Raven. Nat Comput Sci 1:332–336. doi:10.1038/s43588-021-00073-4 38217213

[21] Bouras G, Judd LM, Edwards RA, Vreugde S, Stinear TP, Wick RR. 2024. How low can you go? Short-read polishing of Oxford Nanopore bacterial genome assemblies. Microb Genom 10:001254. doi:10.1099/mgen.0.001254 38833287 PMC11261834

[22] Deatherage DE, Barrick JE. 2014. Identification of mutations in laboratory-evolved microbes from next-generation sequencing data using breseq. Methods Mol Bio 1151:165–188. doi:10.1007/978-1-4939-0554-6_12 24838886 PMC4239701

[23] Tatusova T, DiCuccio M, Badretdin A, Chetvernin V, Nawrocki EP, Zaslavsky L, Lomsadze A, Pruitt KD, Borodovsky M, Ostell J. 2016. NCBI prokaryotic genome annotation pipeline. Nucleic Acids Res 44:6614–6624. doi:10.1093/nar/gkw569 27342282 PMC5001611

[24] Russel J, Pinilla-Redondo R, Mayo-Muñoz D, Shah SA, Sørensen SJ. 2020. CRISPRCasTyper: automated identification, annotation, and classification of CRISPR-Cas Loci. CRISPR J 3:462–469. doi:10.1089/crispr.2020.0059 33275853

